# Strategies to Minimize Antibiotic Resistance

**DOI:** 10.3390/ijerph10094274

**Published:** 2013-09-12

**Authors:** Chang-Ro Lee, Ill Hwan Cho, Byeong Chul Jeong, Sang Hee Lee

**Affiliations:** 1 Department of Biological Sciences, Myongji University, 116 Myongjiro, Yongin, Gyeonggido 449-728, Korea; E-Mails: crlee@mju.ac.kr (C.-R.L.); bcjeong@mju.ac.kr (B.C.J.); 2 National Leading Research Laboratory, Department of Biological Sciences, Myongji University, 116 Myongjiro, Yongin, Gyeonggido 449-728, Korea; E-Mail: guunsung_guy12@hotmail.com; 3 Department of Chemistry, University of British Columbia, Vancouver, BC V6T 1Z4, Canada

**Keywords:** antibiotic resistance, strategy, antibiotic prescribing, antimicrobial stewardship programs, education, hygiene, food animal, new antibiotics

## Abstract

Antibiotic resistance can be reduced by using antibiotics prudently based on guidelines of antimicrobial stewardship programs (ASPs) and various data such as pharmacokinetic (PK) and pharmacodynamic (PD) properties of antibiotics, diagnostic testing, antimicrobial susceptibility testing (AST), clinical response, and effects on the microbiota, as well as by new antibiotic developments. The controlled use of antibiotics in food animals is another cornerstone among efforts to reduce antibiotic resistance. All major resistance-control strategies recommend education for patients, children (e.g., through schools and day care), the public, and relevant healthcare professionals (e.g., primary-care physicians, pharmacists, and medical students) regarding unique features of bacterial infections and antibiotics, prudent antibiotic prescribing as a positive construct, and personal hygiene (e.g., handwashing). The problem of antibiotic resistance can be minimized only by concerted efforts of all members of society for ensuring the continued efficiency of antibiotics.

## 1. Introduction

Antimicrobial agents have been greatly important cornerstones of clinical medicine since the second half of the 20th century and have saved a great number of people from life-threatening bacterial infections. However, the last decade of the 20th century and the first decade of the 21th century have witnessed the emergence and spread of antibiotic resistance in pathogenic bacteria around the World, and the consequent failure of antibiotic therapy, especially in intensive care units (ICUs), which has led to hundreds of thousands of deaths annually [1]. The gradual increase in resistance rates of several important pathogens, including methicillin-resistant *Staphylococcus aureus* (MRSA), vancomycin-resistant *Enterococcus* (VRE), multidrug-resistant (MDR) *Pseudomonas aeruginosa*, imipenem-resistant *Acinetobacter baumannii*, and third-generation cephalosporin-resistant *Escherichia coli* and *Klebsiella pneumonia*, poses a serious threat to public health [2,3,4]. Extended-spectrum β-lactamase (ESBL)-producing pathogens and MRSA are endemic in many hospitals worldwide [5]. Recently, ESBL-producing pathogens and MRSA infections are also increasingly detected in the community (local hospitals managed by local healthcare providers who first treated the patient, as distinguished from specialist hospitals or regional centers equipped with diagnostic and treatment facilities) [6]. Carbapenems are the last stronghold of defense against non-*Enterobacteriaceae* pathogens such as *A. baumannii* and *P. aeruginosa* [5,7]. The increase in carbapenem or fluoroquinolone resistance will be a major threat in the future [8,9]. Recent reports showed that carbapenem-resistant *E. coli* and *Salmonella enterica* are also isolated from food animals [10,11]. Tuberculosis also remains one of the leading public health problems worldwide and its control is hampered by the emergence of MDR *Mycobacterium tuberculosis*, defined as resistance to at least rifampicin and isoniazid, two key drugs in the treatment of the disease [12]. More recent reports described the emergence of extensively drug-resistant (XDR) *M. tuberculosis* that, in addition to being MDR, are also resistant to any fluoroquinolone and to at least one of the three injectable drugs (kanamycin, capreomycin, and amikacin) [12,13]. The hope of overcoming this threat by the development of new antibiotics is diminished both by the decline in novel antibiotic discovery, particularly in the Gram-negative spectrum [14,15,16,17], and by the possibility that pathogens will evolve resistance to novel antibiotics just as they adapt quickly to existing antibiotics [1,18,19]. Therefore, to address the problem of antibiotic resistance, effective strategies are required. This review will summarize and discuss various strategies to minimize antibiotic resistance.

## 2. Appropriate Antibiotic Prescribing

Since the resistance to the first commercial antimicrobial agent (penicillin) was identified in 1948 [20], almost every known bacterial pathogen has developed resistance to one or more antibiotics in clinical use [5]. As antibiotic-resistant pathogens are observed almost concurrently with the use of new antibiotics in hospitals [21], one can easily suppose that wherever antibiotics are used, antibiotic resistance will inevitably follow. Unfortunately, although antibiotic resistance has increased, the development of novel antimicrobial agents has dramatically declined over the past 30 years [18]. Therefore, to prevent the return of the pre-antibiotic era, one must use existing antibiotics more judiciously.

**Figure 1:**
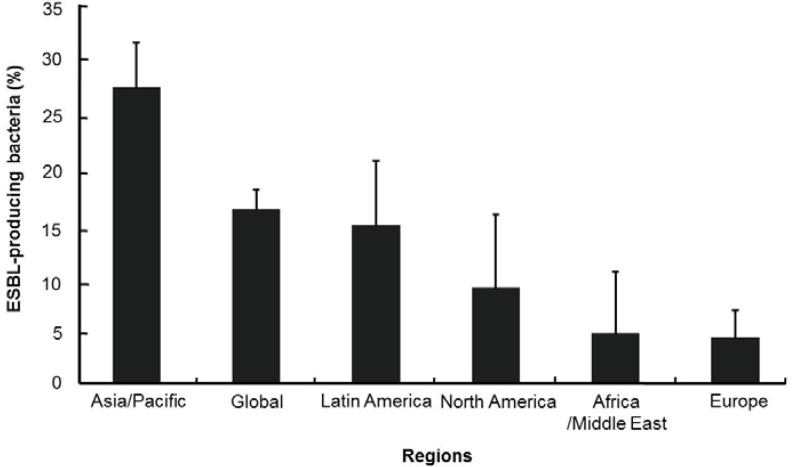
Regional proportions (%) of ESBL-producing bacteria (*Escherichia coli*, *Klebsiella pneumonia*, *Klebsiella oxytoca*, and *Proteus mirabilis*) isolated from patients with appendicitis: 2008–2010 results from the study for SMART with 95% confidence intervals.

**Figure 2:**
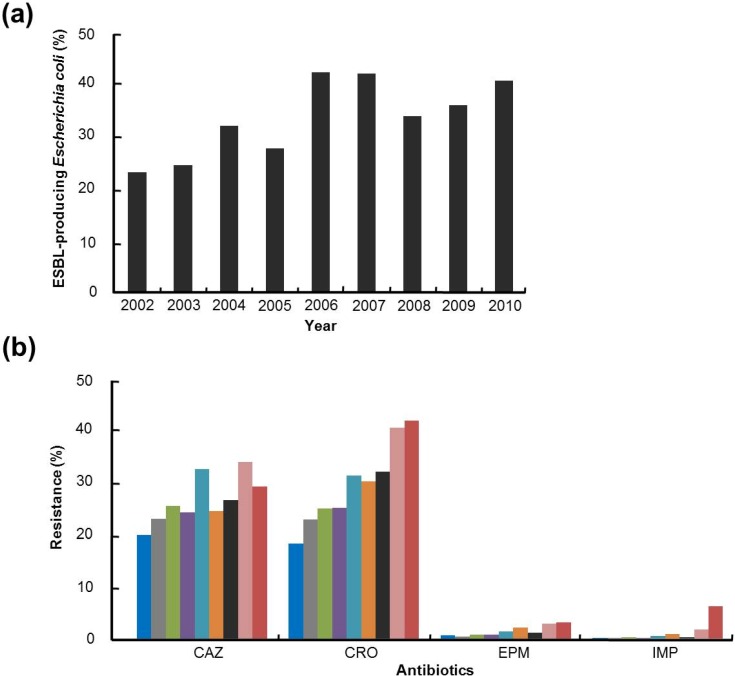
(**a**) ESBL-producing rates (%) in *Escherichia coli* isolated from intra-abdominal infections in the Asia-Pacific region from 2002–2010 results from the study for SMART. (**b**) Resistance rates (%) for various antibiotics (CAZ: ceftazidime; CRO: ceftriaxone; EPM: ertapenem; IMP: imipenem) in Enterobacteriaceae isolated from intra-abdominal infections (IAIs) in the Asia-Pacific region from 2002–2010 (2002, blue; 2003, gray; 2004, green; 2005, purple; 2006, sky-blue; 2007, orange; 2008, black; 2009, pink; 2010, red) results from the study for SMART.

**Figure 3:**
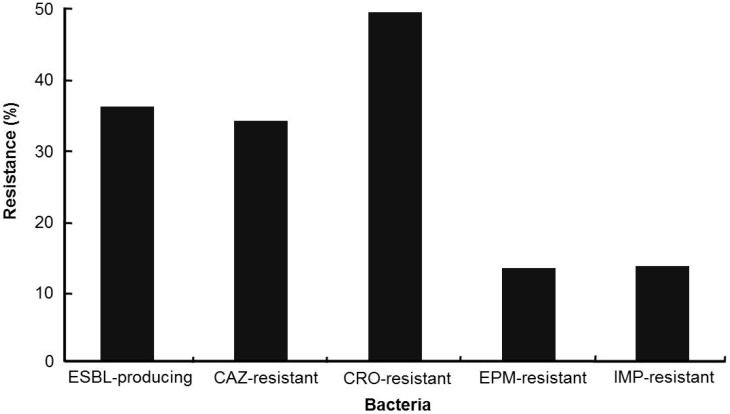
Resistance rates (%) for various antibiotics (CAZ, ceftazidime; CRO, ceftriaxone; EPM, ertapenem; IMP, imipenem) in *Enterobacteriaceae* isolated from urinary tract infections (UTIs) in the Asia-Pacific region from 2009–2010 results from the study for SMART.

The Study for Monitoring Antimicrobial Resistance Trends (SMART) is the premier global surveillance system on antimicrobial resistance of microbes. Data from SMART studies show that the level of antimicrobial resistance differs by geographic region and is highest in Asia-Pacific conutries, like in the case of patients with appendicitis (Figure 1) [22,23]. Latest results from the SMART study also showed that the ESBL-positive rates in *E. coli* isolated from intra-abdominal infections (IAIs) in the Asia-Pacific region almost doubled between 2002 and 2010 to 40.8% (Figure 2(a)) [24,25,26,27,28]. The large increase in ESBL producers, after the early 2000s, has been a major problem when it comes to treating infections due to *Enterobacteriaceae* [15,29]. Another important issue in *Enterobacteriaceae* is its resistance to expanded-spectrum cephalosporins, the effective antibiotics used to treat enterobacterial infections. Figure 2(b) shows a gradual increase in resistance to expanded-spectrum cephalosporins (ceftazidime and ceftriaxone) in *Enterobacteriaceae* obtained from patients with IAIs in the Asia-Pacific region between 2002 to 2010 [24,25,26,27,28,30]. The upsurge in enterobacterial stains, which are resistant to carbapenems, such as imipenem and ertapenem, is particularly a threat to the successful treatment of enterobacterial infections, in addition to being a cause for lack of drugs for antibiotic-resistant Gram-negative pathogens (Figure 2(b)). The proportion of carbapenem-resistant *Enterobacteriaceae* isolates increased sharply between 2002 and 2010. The prevalence of imipenem resistance rose sharply from 0.2% to 6.3% and ertapenem resistance also significantly increased from 0.6% to 3.1%. SMART’s studies on urinary tract infection (UTI) in the Asia-Pacific region began in late 2009 [31]. Data from the studies carried out in 2009–2010 reveal that the proportion of urinary *E. coli* exhibiting ESBL-producing phenotype is 36% (Figure 3), which is similar to that of ESBL-producing *E. coli* isolated from IAIs in 2009 (Figure 2(a)) [31,32]. However, the proportions of enterobacterial stains resistant to carbapenems such as imipenem and ertapenem were about 2–4-fold higher in patients with UTIs (Figure 3) than in patients with IAIs (Figure 2(a)). Resistance to expanded-spectrum cephalosporins was also higher in terms of enterobacterial stains obtained from patients with UTIs.

Generally, mortality from bacteremia is significantly higher with ESBL producers than with non-producers [33]. As carbapenems are not hydrolyzed by ESBLs, carbapenem (primarily imipenem) use was associated with significantly low 14-day mortality (8%) in patients with *Klebsiella* bacteremia, as compared to 25% with quinolones, 50% with cefepime, and 67% with piperacillin/tazobactam [34]. In a recent study of 244 patients with bacteremia by ESBL-producing *E. coli* or *K. pneumonia*, a 30-day mortality was found to be greater in noncarbapenem-treated patients than in carbapenem-treated patients, whereas ertapenem therapy was found to be more effective than imipenem or meropenem therapy [35]. However, because of the upsurge in enterobacterial stains resistant to carbapenems, Paterson suggested that carbapenems must be used wisely if there are the drugs of choice for the treatment of IAIs and UTIs caused by ESBL-producing *Enterobacteriaceae* [34,36]. For uncomplicated IAIs such as unperforated appendix or gall bladder surgery, the use of carbapenems is not recommended. However, for more complicated cases, where surgery is not enough to cure the infection, ertapenem (the Group 1 carbapenem) is the first-line treatment and meropenem, imipenem, or doripenem (the Group 2 carbapenems) as the next one in case of severe sepsis and ICUs [36]. For patients who cannot use carbapenem, he recommends tigecycline.

As in the case of IAIs with ESBL producers, many guidelines for appropriate antibiotic prescribing have been published [37,38,39,40,41,42,43,44]. However, those guidelines need to be continually updated based on the advances in knowledge. In community-acquired pneumonia (CAP), hospital-acquired pneumonia (HAP), ventilator-associated pneumonia (VAP) and healthcare-acquired pneumonia (HCAP), an adequate understanding of each type of infection can provide physicians with more informed choices of antibiotics and thus, help in preventing the development of resistance. Guidelines for the management of adults with nosocomial pneumonia (HAP, VAP, and HCAP) were published in 2005 [39]. A recently published study suggests that compliance with the guidelines can increase mortality in HAP/HCAP patients [45]. Out of 303 patients, who were at the risk for multidrug-resistant pneumonia, 129 patients were treated by guideline-compliant prescription and 174 patients by non-compliant prescription. Between these two groups, the 28-day mortality was 34% in the compliance group and 20% in the non-compliance group. This suggests the requirement of upgraded guidelines for the management of adults with nosocomial pneumonia. Studies of optimized treatment for VAP were also recently carried out [46,47]. Three-hour infusions of cefepime 2 g every 8 h or meropenem 2 g every 8 h plus tobramycin and vancomycin reduced infection-related mortality by 69% (8.5% *vs*. 21.6%), and shortened infection-related length of stay by 55% (11.7 *vs*. 26.1 days), as compared to the outcomes of historical controls using the traditional low dosing regimen. The number of superinfections observed was also reduced [46,47]. This indicates the benefits of high doses of antibiotics in prolonged infusions. In HAP patients, it was reported that early administration of multi-antibiotic therapy can improve survival rate as compared to what is possible using mono-antibiotic therapy [48]. Some studies showed that in comparison to CAP patients, HCAP patients have more severe illness, higher mortality for moderate severity, higher frequency comorbidity, worse functional status, and more inappropriate antibiotic prescribing [49,50]. Therefore, appropriate initial antibiotic therapy should be treated to reduce mortality of HCAP patients. Niederman recommends the use of ertapenem for both severe and non-severe HCAP patients with no risk of *P. aeruginosa*, and imipenem or meropenem for HCAP patients with *P. aeruginosa* [36,51]. As in cases of IAIs and nosocomial pneumonia, adherence to guidelines for appropriate prescribing of effective antibiotics (e.g., carbapenems) can minimize the selection of new resistance.

## 3. Antimicrobial Stewardship Programs

Many institutions conduct Antimicrobial Stewardship Programs (ASPs) to optimize antimicrobial therapy, reduce treatment-related cost, improve clinical outcomes and safety, and reduce or stabilize antimicrobial resistance [52]. The formal guidelines for ASPs were developed in 2007 by the Infection Diseases Society of America (IDSA) and the Society of Healthcare Epidemiology of America (SHEA) [40]. Typically, ASPs are executed by multidisciplinary antimicrobial utilization teams comprising physicians, pharmacists, microbiologists, epidemiologists and infectious disease specialists, with adequate experience in their respective fields. Many studies demonstrated that ASPs have the potential to restrict the emergence and spread of resistance [53]. ASPs have demonstrated a link between antimicrobial use and the emergence of resistance. The following are some examples in this regard: fluoroquinolone use and MRSA [54]; vancomycin use and vancomycin-resistant enterococci [55]; cephalosporin use and cephalosporin-resistant *Enterobacteriaceae* [56]; and carbapenem use and carbapenem-resistant *Acinetobacter*, *Pseudomonas*, and *Enterobacteriaceae* [57,58]. Recent research demonstrated that restricting ciprofloxacin use improve susceptibility of *P. aeruginosa* to the Group 2 carbapenems, such as imipenem or meropenem [59]. A reduction by 90% in the use of ciprofloxacin between 2000 and 2010 led to a concurrent reduction (25% to 10–15%) in the proportion of carbapenem-resistant *P. aeruginosa*, although carbapenem use increased from 12 daily defined doses (DDDs)/1,000 patients in 2004 to 28 DDDs/1,000 patients in 2010 [59].

ASPs are based primarily on education, coupled with the “front-end” interventions (e.g., restricting the availability of selected antimicrobial agents) or the “back-end” interventions (e.g., reviewing broad-spectrum empirical therapy and then streamlining or discontinuing therapy on the basis of antimicrobial susceptibility testing (AST) results and clinical response) [60,61]. In the “front-end” interventions, the following aspects can be included: (i) the development of situation-specific treatment guideline; (ii) education of prescribers; (iii) AST; (iv) accurate organism-identification; (v) understanding pharmacokinetic (PK) and pharmacodynamic (PD) properties of drugs which helps selecting optimal dose and duration of antibiotics; (vi) minimizing the effect of antibiotics on the microbiota and host immune homeostasis; and (vii) formulary restriction and preauthorization. Formulary restriction and preauthorization is particularly considered as the main strategy in IDSA/SHEA guidelines [40] and several studies show that this strategy has been proven successful in reducing the use of antibiotics and pharmacy cost [60,62,63,64].

Understanding PK and PD properties of antibiotics is also an important factor [46,47]. Fluoroquinolone is one of the most commonly prescribed antibiotics in hospitals. In 1976, Stamey *et al*. demonstrated, for the first time, the direct relationship between antibiotic underdosage and the emergence of antibiotic resistance [65]. They showed a directed increase in the number of nalidixic acid-resistant strains with decreasing concentration of nalidixic acid. Since this pioneering study, similar results have been reported, and now, it has been generally accepted that the use of antibiotics at low concentrations over long periods is an optimal way to enrich resistant bacterial populations [66]. For determining the optimal dosing regimen with minimal induction of resistance, the two useful points of reference are minimum inhibitory concentration (MIC) and mutant prevention concentration (MPC) [67]. Emergence of resistance occurs within in the frame of a selective compartment, termed as the mutant selection window (MSW): the lower boundary corresponds to the MIC of the susceptible cells, whereas the upper boundary, the MPC, restricts the growth of the entire population including the resistant mutants. By careful use of PK/PD concepts and the MPC strategy, the effectiveness of antibiotics can be optimized and the selection of resistant mutants limited. Emergence of a resistant bacterial subpopulation within a susceptible wild-type population can be restricted with a regimen using an antibiotic dose that is sufficiently high to inhibit both susceptible and resistant bacteria. Although the MSW has been determined for many antibiotics [68,69], the data available continue to be inadequate, and further work is needed to characterize these target drug concentrations fully.

Antimicrobial susceptibility testing (AST) can also be an important aid for a rapid and reliable prediction of antimicrobial success in the treatment of bacterial infections. The most conventional methods are the ones that detect phenotypic resistance by measuring bacterial growth in the presence of the antibiotic being tested [70]. Although these standard phenotypic resistance tests are highly sensitive to detection of resistance, they require rather large numbers of viable cells, limited organism spectrum, complicated pre-analytical processing, analytical variability, long time to obtain results, and high cost [71]. In recent years, novel approaches for the rapid detection of resistance in bacterial pathogens are developed. Included among these are the PCR-based techniques, mass spectrometry, microarrays, microfluidics, flow cytometry, isothermal microcalorimetry (IMC), cell lysis-based approaches and whole-genome sequencing, whose ability to detect resistance in various bacterial species has been demonstrated [70,71]. However, it remains to be determined whether these novel methods can achieve sufficient sensitivity and specificity as compared with those of the classical phenotypic methods to justify their use in routine clinical practice [70]. Rapid and accurate organism-identification also benefits the patient and the effectiveness of ASPs. For rapid identification of *Staphylococcus* species, *Enterococcus faecalis*, *Enterococcus faecium*, *E. coli*, *K. pneumoniae*, *P. aeruginosa*, *Clostridium difficile*, and *Candida* species from clinical samples, molecular diagnostic methods have been developed [72].

Over the past few years, remarkable advances in next-generation sequencing technology have enabled elaborate molecular characterizations of the microbial ecosystem, and recent reports using this technology have shown that the microbiota substantially affects human health and physiological development, including nutritional processing, prevention of pathogen invasion, host development, and maturation and homeostasis of immune system [73,74,75,76]. Antibiotic treatment to remove pathogens is likely to cause both short-term and long-term impacts on the commensal microbiota and this disturbance in the microbiota can trigger both transient and persistent changes in host immunity and physiology [12,13]. Therefore, when prescribing antibiotics for patients, physicians should be aware of the effects that antibiotics have on the patient’s microbiota. Like cases of ciprofloxacin [77] and vancomycin [78], antibiotic treatment typically causes a dramatic and immediate decrease in the phylogenetic diversity of the previously stable microbiota, but the microbiota begins to recover and resembles the pretreatment state within days or weeks after the antibiotic have been removed. But, some members, such as low-abundance members of the community, are not fully recovered within 6–10 months [79].

Studies using animal models have demonstrated that different antibiotic therapies cause distinct effects on the microbiota. For instance, treatment with a combination of amoxicillin, metronidazole, and bismuth could be rapidly recovered to pretreatment levels after treatment withdrawal, while treatment with the broad-spectrum antibiotic cefoperazone produced persistent decrease in the overall phylogenetic diversity during six weeks after removal of the treatment [78,80]. Interestingly, bacteria that are affected by antibiotic treatment are not limited to those which are directly susceptible to antibiotic. Despite the antimicrobial activity of vancomycin being restricted to Gram-positive bacteria, some Gram-negative bacteria were significantly reduced [78]. This phenomenon is partly caused by the dysregulation of host immune homeostasis as a consequence of changes in the microbiota [81]. Recent many reports have shown antibiotic-associated changes in host immunity: reduced expression of REG3γ by broad-spectrum combination antibiotic treatment (vancomycin, neomycin, and metronidazole) [12], reduced expression of REG3β by streptomycin therapy [82], depletion of T helper 17 cells by vancomycin therapy [83], reduced production of RELMβ by ampicillin therapy [84], reduced expression of TLR2 and TLR4 in peritoneal macrophages by the treatment of streptomycin and cefotaxime [85], and disrupted mucus layer by metronidazole therapy [86]. Besides effects on host immune system, repeated antibiotic exposure of the microbiota increased an abundance of the antibiotic resistome and it could persist for long periods even after removal of the treatment, through the horizontal gene transfer of antibiotic resistance determinants [79,87,88,89,90]. In summary, antibiotics can give stable and lasting alterations to the gut microbiota, reducing mutualistic benefit by the host-microorganism associations. As eliminating the need for antibiotics is impossible at this time, further studies of how each antibiotic affects the microbiota and host immune system are required. To minimize these effects, some strategies such as bacteriotherapy [73], probiotic intervention [91], the administration of TLR agonists (e.g., flagellin) during antibiotic therapy [92], are developed.

In the “back-end” interventions, the following aspects can be included: (i) clinical decision support; (ii) post-prescription review and feedback; (iii) the development of protocols for de-escalation of therapy on the basis of AST and clinical response; (iv) diagnostic testing using biomarkers such as procalcitonin or C-reactive protein (CRP); (v) determination of therapy length; and (vi) antibiotic heterogeneity (cycling and mixing). Post-prescription review and feedback is one of the cornerstones of ASPs [40]. Although the therapy can be continued indefinitely, at the discretion of the treating clinician, for 48–72 h after initial antimicrobial prescription, post-prescription review by multidisciplinary antimicrobial utilization teams is required to either modify or discontinue treatment, depending on the clinical responses and guidelines; also, appropriate feedback can be provided to prescribers in order to modify or discontinue therapy [93]. Several studies demonstrated useful outcome from post-prescription review and feedback strategy [63,93,94,95]. Interestingly, the outcome differed across institutions and it benefited only those hospitals that have well-established ASPs [95]. Diagnostic testing using biomarkers can also reduce antibiotic consumption. Serum levels of procalcitonin and calcitonin precursor increase dramatically through moderate-to-severe inflammation by bacterial infections, but remain at comparatively lower levels in viral infections and nonspecific inflammatory diseases [41,96]. Many studies show that the use of procalcitonin, as a guide to discontinue treatment with antibiotics, can significantly reduce antibiotic consumption, particularly in patients with respiratory tract infection in the community and in critically ill patients in the ICUs [41,97,98]. But, accurate cutoff values of procalcitonin levels to continue or discontinue the use of antibiotic should be arranged and the cost of diagnostic testing using procalcitonin is also needed to be evaluated.

Antibiotic heterogeneity, such as antibiotic cycling (also known as “antibiotic rotation”) or antibiotic mixing (also known as “antibiotic diversity”), continues to be a debatable subject, although many investigators have studied its effects on antibiotic resistance with the help of clinical investigations or theoretical models [19,99,100,101,102]. Antibiotic cycling involves an exchange of one class of antibiotics with those of a different class possessing a similar spectrum of activity, but different mechanisms of antibiotic resistance (e.g., β-lactams, aminoglycosides, and fluoroquinolones) [99]. In theory, it seems that such strategies can strongly block the establishment of a stable resistant population, but clinical evidences do not vindicate the modeling predictions [103,104,105,106,107]. In 2004, mathematical modeling suggested that antibiotic cycling is unlikely to reduce antibiotic resistance, and that antibiotic mixing may prove to be more effective [108].

However, other reports in 2010 argue that antibiotic cycling is likely to select optimally against antibiotic resistance in theory [109,110]. In fact, recent reports show that improved cycling strategy can reduce antibiotic resistance [111,112,113,114,115,116]. Periodic Antibiotic Monitoring and Supervision (PAMS), in particular, is a novel strategy that is based on antibiotic heterogeneity [99,101,116]. Prescribing decisions are supervised by a multidisciplinary antimicrobial stewardship team comprising two infectious disease physicians, a pharmacist and an infection control nurse. During PAMS, the “recommended”, “restricted” and “off-supervision” classes of antibiotics were changed every 3 months, depending on antibiotic use in the preceding period and the incidence of resistance. PAMS is a real-time antibiotic cycling strategy; so it decides on the “recommended”, “restricted” and “off-supervision” classes of antibiotics based only on the on-going results of the study, and not at the beginning of the study. The results obtained by using PAMS show that this strategy is significantly successful [116]. Its success depends probably on real-time monitoring of the incidence of resistance; thus, for the successful execution of these complex strategies, the development of effective and robust monitoring methods and quality supervision may be required [99]. Although a few reports demonstrated the usefulness of the strategy of antibiotic mixing [46,47,117,118,119], many more studies are required to demonstrate the effectiveness of antibiotic heterogeneity (cycling and mixing). It is noteworthy that antibiotic mixing can carry the risk of inappropriate usage of antibiotics because of the open formulary, whereas antibiotic cycling can increase the duration of antibiotic treatment.

## 4. Education

Much of the success of ASPs depends on educating the clinicians, especially on making their everyday treatment decisions [120]. It is noteworthy that almost any clinician can prescribe antibiotics without any regulation or certification, whereas only specialists in oncology can prescribe and administer anti-cancer drugs [52]. To optimize antimicrobial prescribing, the prescribers should have appropriate knowledge of general medicine, microbial virulence, immunological and genetic host factors, PK and PD properties of drugs, and basic knowledge of epidemiology. Prescribers of antibiotics such as physicians and pharmacists encounter dual, somewhat contradictory responsibilities. On the one hand, they want to provide optimal therapy for their patients and this responsibility tends to promote an overuse of antibiotics. On the other hand, they have a responsibility to future patients and to public health in sustaining the efficiency of antibiotics and minimizing antibiotic resistance, but this responsibility is sometimes ignored. There have been reports that about 50% of the antibiotic prescriptions, both in the community and in hospitals, can be considered inappropriate (inadequate dosing and wrong duration) [40,121]. As most of the antimicrobial agents are used in primary care [122,123], education on antibiotic prescribing in primary care is important. Some reports demonstrate that, notwithstanding the advice to decrease antibiotic prescribing in primary healthcare, misuse or overuse of antibiotics continues [124,125]. In France, only 21% of the primary care physicians followed the guidelines in prescribing antibiotic treatment for urinary tract infection [125]. About 50% of primary care prescriptions for nephritis were wrong, and 70% of asymptomatic bacteriuria was treated with antibiotics. Similar results were reported from Greece. Only 55% of the community physicians restricted antibiotic use for sore throat, and only 26% utilized a strep-test to guide antibiotic use. Eighty-nine percent of the physicians prescribed antibiotics for chronic obstructive pulmonary disease exacerbations and only 17% of them followed the widely accepted Anthonisen criteria [124].

Lack of knowledge in microbial virulence and antibiotics may significantly affect the quality of prescribing. Physicians with inadequate knowledge may prefer prescribing maximal broad-spectrum treatment. Therefore, educating physicians is certainly required. Recent reports emphasize that undergraduate training courses would be successful if the students are imparted with adequate knowledge, and trained in developing the right attitude and behavior [126]. As is the practice in some countries, including the UK and Scotland, education on prudent antibiotic prescribing should be included as a component of the undergraduate curriculum [37,127]. Teaching postgraduate students, particularly the prescribers of antibiotics in the community, requires internship/foundation training or close collaboration between local healthcare providers and academicians. The teacher, who offers the guidelines for antibiotic treatment, must also be trained on the available educational strategies, as also the current information on antimicrobial stewardship [126]. As has been the practice in some countries, nurses, clinical pharmacists, and midwives may also be allowed to prescribe some antibiotics in special clinical situations [127,128,129]; all healthcare professionals who have to deal with patients must be educated about prudent antibiotic treatment and management of patients demanding an overuse of antibiotic. Antibiotic management requires effective teamwork between all healthcare professionals. If patients receive inconsistent messages from healthcare professionals when taking antibiotics, all efforts of prudent antibiotic prescribing may become unsuccessful. Therefore, all healthcare professionals must receive continual education on prudent antibiotic prescribing [129].

Because of serious misunderstanding in elementary knowledge on antibiotic use (e.g., antibiotics are useful for colds), many programs have been made for teaching children in Europe and the United States (U.S.) [43,126,130]. But, most of the curricula of these countries include only the topic of human health and hygiene, whereas information about antibiotics and their prudent use is rather scanty. Therefore, all children belonging to the 9–11-year-old or 13–15-year-old age group, who need to undergo compulsory education, are the most appropriate target for education on antibiotics and their prudent use [126,130]. In 2007, the Advisory Committee on Antimicrobial Resistance and Healthcare-Associated Infection (ARHAI) was established to educate both healthcare professionals and the public [131]. The public education subgroup took on the public-facing antimicrobial campaigns, which comprised posters with a positive message on how the public could help themselves when they have a cold [131,132]. These public campaigns can induce in outpatients the habit of more prudent use of antibiotics, especially in high-prescribing countries [133]. But, for an effective education of patients and the public, the role of professionals is very important. The professionals must give the public clear information about the duration of symptoms, self-care, benefits and limitations of antibiotics, and antibiotic resistance.

## 5. Hygiene and Disinfection

MDR pathogens often cause hospital-acquired infections, which require more expensive antibiotics and further hospitalization. In the United States (U.S.), 1.7 million hospital-acquired infections are recorded each year, which result in about a hundred thousand deaths [134,135]. Although the main source of MDR pathogens is thought to be the endogenous flora of patients, healthcare workers are also considered an important source [67,136]. Therefore, appropriate hospital disinfection and personal hygiene of healthcare workers are required to prevent hospital-acquired infections. The Centers of Disease Control and Prevention (CDC) and the SHEA offered guidelines for preventing nosocomial transmission of MDR bacteria in hospitals [44,136]. Transmission of healthcare-associated pathogens through the hands of healthcare workers is particularly the most common cause for spreading [137,138]. Contamination of the hands of healthcare workers could result either directly from contact with patients or indirectly from touching contaminated environmental surfaces [135,139]. Several studies have demonstrated that an increase in handwashing compliance significantly decreases nosocomial infections by MRSA in intensive care units (ICUs) [140,141]. The World Health Organization (WHO) and the CDC presented hand hygiene guidelines in healthcare [142].

Although there has been convincing evidence that compliance with hand hygiene recommendations can reduce hospital-acquired infection rates [142,143,144,145,146], such compliance by healthcare workers remains low worldwide, in both developed and developing countries [137,142]. Although hand hygiene practices are simple, compliance with those practices requires bringing about a change in human behavior, which is affected by complex factors, such as attitudes and beliefs [147,148]. This renders many public health campaigns worldwide unsuccessful. Therefore, when designing studies to investigate compliance, a close collaboration with behavioral and social sciences is required [149]. A recent report shows that positive role modeling, such as hand hygiene behavior of senior practitioners, considerably improves compliance with hand hygiene requirements [150]. It is worth noting that wearing gloves can prevent hands from becoming contaminated with pathogens [151], but it cannot be a substitute for hand hygiene, because of the possibility of contamination during the process of removing the gloves [152,153]. Despite the controversy over issues relating to the use of alcohol-based hand rubs and spread of spore-forming pathogens, such as *Clostridium difficile* [154,155], several studies demonstrated that the relationship between alcohol-based hand rubs and the incidence of clinical isolates of *C. difficile* is weak [156,157]. Therefore, further studies on the commonly used agents of hand hygiene, including alcohol, chlorohexidine, chloroxylenol, iodine, triclosam, and octenidine [158], are required.

In addition to hand hygiene, gloves, gowns, uniforms, and plastic aprons should also be considered. Many studies have demonstrated that the gloves or gowns of healthcare workers can be colonized with MDR pathogens, such as MRSA or VRE [159,160,161]. And, environmental cleaning in hospitals is also associated with a reduction in the transmission of healthcare-associated pathogens, including MRAS, VRE, and *C. difficile*, and *Acinetobacter* species [135,162,163]. Several reports show that improved environmental cleaning would result in decreasing the extent of patient-to-patient transmission of MRSA or VRE [164,165,166]. The CDC and Hospital Infection Control Practices Advisory Committee presented guidelines for environmental infection control and sterilization in healthcare facilities [167]. As *C. difficile* and *A. baumannii* can survive both on dry surfaces and in water for prolonged periods (from weeks to months) [168,169,170], the recent guidelines by the CDC and the SHEA recommend the use of 1:10 dilution of sodium hypochlorite for environmental cleaning [171]. There is increasing evidence that hand-touch sites are habitually contaminated by MDR pathogens [164,172,173,174]. This is because cleaners focus their attention commonly on general surfaces such as floors and bathrooms, and easily ignore hand-touch sites [165,175]. Therefore, prioritizing the cleaning of high-risk hand-touch sites would be the most cost-effective cleaning strategy [164].

## 6. Veterinary Medicine

Antibiotics have also been used in veterinary medicine since the first commercial antibiotic, penicillin, became available for the treatment of human diseases [176]. Although some antibiotics are designed exclusively for veterinary use, most of the antibiotics being used in veterinary medicine belong to the same antimicrobial classes as those being used for human diseases [5,177]. Antibiotics are administered to food animals in agriculture worldwide as veterinary medicine and as growth-promoting agents to obtain sufficient amount of food [178,179]. Surprisingly, until recently, about 70% of the antibiotics administered to food animals was for non-therapeutic purposes, such as growth promotion [180,181,182,183]. Such usage is generally performed through feeding at very low concentrations over long periods (a very dangerous practice which could enrich resistant bacterial populations) [66,184]. In the 1990s, some reports have shown that the growth-promoting antibiotic avoparcin, a member of the same glycopeptides family as that of vancomycin, leads to the selection of vancomycin-resistant *Enterococcus faecium* [185]. And low levels of VRE are found in fecal samples of food animals or humans outside hospitals, in countries where avoparcin has never been used (e.g., Sweden and the U.S.) [185,186,187]. In the EU, the use of avoparcin was banned in 1997 to preserve vancomycin’s clinical utility. Even though the use of antibiotics for growth promotion in the feed of food animals was totally banned in the EU and a similar ban is being contemplated in the U.S. [5,188], medically important antibiotics are still being fed routinely to food animals to promote growth and to ward-off potential bacterial infections in the stressed and crowded livestock and aquaculture environments [5,182]. Recent reports about antibiotics, which is critically important to human therapy, are frightening, in that they report the presence of ESBL-producing and carbapenemase-positive *Enterobacteriaceae* strains in food animals, and MRSA in various food animal species and food products [10,11,189,190], as well as plasmid-mediated quinolone resistance in food animals and food products [191].

As global production of aquatic species (fish, shellfish, shrimp, and molluscs) has been growing rapidly in the last decade, the use of antibiotics in aquaculture also increased [5,182]. Many MDR fish pathogenic bacteria were found in fish farms [192,193]. In addition, it has been shown that multi-resistance plasmids from some fish pathogens, such as *Aeromonas salmonicida*, can be transferred to human pathogens such as *E. coli* [194]. The amount of antibiotic use in plants is generally low as compared that in humans and food animals. Of the total antibiotic use in the U.S., about 0.1% was estimated to have been used in plant agriculture [195]. However, because spraying antibiotics in the open environment might increase the emergence of MDR bacteria, the careful concern is required.

Because stress in crowded environments weakens the immune system in food animals and antibiotics can prevent bacterial infections, antibiotics are considered useful for intensive breeding of animals. But, the use of antibiotics in veterinary medicine, agriculture, and aquaculture needs to be reduced. If the introduction of resistance genes into human through food animals is not restricted, the problem of antibiotic resistance in human medicine will not be overcome. Therefore, to prevent the emergence and transfer of antibiotic resistance in food animals, new methods to manage infectious diseases in animal husbandry are required. For example, optimal use of existing vaccines can be a viable alternative [196]. Improving hygiene [197], using enzymes, probiotics, prebiotics, and acids to improve health [198,199], and utilizing bacteriocins, antimicrobial peptides, and bacteriophages as substitutes for antibiotics might be good methods to promote growth in food animals and decrease infectious diseases in them [200,201]. Further, it is worthwhile to formulate internationally acceptable standard protocols about the use of antibiotics in animal husbandry and about surveillance programs to monitor global emergence of MDR bacteria.

## 7. The Development of Novel Antibiotics

**Figure 4:**
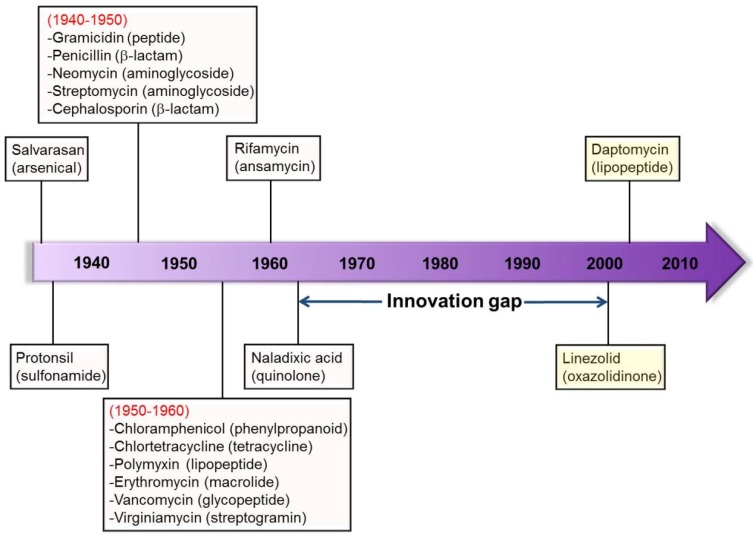
The emergence of novel antibiotics.

Antimicrobial drugs such as antibiotics are a unique class of drugs that does not directly target human biochemical processes but instead affect the growth of invading pathogens and commensal microbiota. Bacteria can easily adapt to their environmental changes and decrease their susceptibility to antibiotics by several mechanisms, including mutation and horizontal gene transfer within and between species [202]. Therefore, new weapons are always indispensable for combating bacterial infections. Nevertheless, most of the antibiotic classes being used today were discovered during the period 1930–1960. Besides, during the past 30 years, only two new systemic classes of antibiotics (oxazolidinones in 2000 and cyclic lipopeptides in 2003) and two topical classes (pseudomonic acids in 1985 and pleuromutilins in 2007) were introduced in the market (Figure 4) [18,203].

Even so, neither of these new systemic classes can effectively act against Gram-negative bacteria, in which MDR is an acute problem and the treatment options are limited [15,16,17,204]. Unlike Gram-positive bacteria, Gram-negative bacteria have an additional outer membrane comprised of lipopolysaccharide (LPS), which offers an additional barrier to block the invasion of antibiotics [205]. 

Recent report shows that only compounds with molecular weight (MWs) of less than 600 Da can penetrate the outer membrane of Gram-negative bacteria, whereas compounds with MWs of more than1,000 Da (e.g., vancomycin of 1,449 Da and daptomycin of 1,620 Da) can pass through the Gram-positive bacteria cell wall [206].A limited number of antibiotics targeting Gram-negative bacteria, such as polymyxin/colistin and azithromycin with high MWs, can penetrate the outer membrane of Gram-negative bacteria using active transport mechanisms that facilitate transport through the outer membrane. Besides these technical obstacles, another obstacle to developing novel antibiotics is the economic feasibility. Large pharmaceutical companies want to obtain an annual turnover of about $1 billion for their broad-spectrum drugs with wide usage [19]. Even if wide usage is achieved, those antibiotics may become short-term drugs, unlike drugs for heart disease, Alzheimer’s disease, or arthritis, because of the emergence of resistance [207]. In addition, sales of a novel drug may be severely affected by the wide use of cheap generic antibiotics, particularly in the community [203]. Besides, for clinical development, the US Food and Drug Administration (FDA) and the European Medicines Agency (EMA) may want drugs, which are superior, rather than equivalent, to the reference antibiotic [208]. Therefore, new business models and political actions are required. Such political actions could include innovative financial support, such as offering subsidies, reducing financial and transactional costs of research and development (R&D) process, and introducing outcome-based rewards [209]. Tax breaks, simplification of clinical trial requirement, sharing research and development costs with big pharmaceutical companies, and sharing compound libraries involved in the discovery of antibiotics could also invigorate the antibiotic development of pharmaceutical companies [210]. The IDSA has recently launched the “10 × 20” initiative aiming at the development of 10 new, safe and effective antibiotics by 2020 [203,211]. The EU has also made an effort to develop innovative incentives for effective antibiotics [212,213].

Recent reports on antimicrobial agents being developed show that there are approximately 70 new active substances in clinical development with activity against MDR pathogens [14,203,214,215]. Some of these new active substances can be systemically administered and are assessed to have either a new mechanism of action or a new target. Although, based on actual data, four of them are known to have an activity against MDR Gram-negative bacteria, not a single agent has any new mechanism of action. This reflects the current lack of development of agents against MDR Gram-negative bacteria with new action mechanisms. A recent report argues that, possibly, major suitable targets of antibiotics have already been identified [216]. However, some novel classes of agents against MDR Gram-positive pathogens, such as MRSA, are currently in diverse stages of development and are undergoing clinical trials [214]. To discover new classes of antibiotics, novel strategies for rational design and screening-based approaches are required. Unlike conventional antimicrobial drugs, new strategies are also presented for the treatment of microbial diseases, such as host defense peptides, bacteriophages, vaccines, immunoglobulins, and probiotics [217]. 

## 8. Conclusions

Jawetz’s opinion about antibiotics, as expressed in his manuscript published in 1956, reads: “on the whole, the position of antimicrobial agents in medical therapy is highly satisfactory. The majority of bacterial infections can be cured simply, effectively, and cheaply. The mortality and morbidity from bacterial diseases have fallen so low that they are no longer among the important unsolved problems of medicine. These accomplishments are widely known and appreciated.” [218]. In those days, Jawetz was unaware of the enormous ability of bacteria to adapt easily to new environments, such as the exposure of antibiotics. But, more serious than his opinion is the problem that, even now, quite a few people including the public, politicians, and some prescriber, harbor partly a similar opinion. Even though treatments of most human diseases have improved over time, the treatment conditions for bacterial infections have gradually worsened, because of antibiotic resistance and the lack of new drugs. Antibiotic resistance has gradually increased over the past two decades and is now widespread all over the world. Although antibiotic resistance is not eliminated, it can be controlled to prevent a return to the pre-antibiotic era. Apparently, it is now clear that antibiotic use can increase the emergence of antibiotic-resistant bacteria, and reducing prescribing is one of the effective ways to reduce selection pressure. To prevent overuse and misuse of antibiotics, a formalized, practical guideline for appropriate antibiotic prescribing should be developed and followed by formulary implementation of the guidelines contained therein. However, the development of quick, effective molecular diagnostic techniques for identification and epidemiological surveillance of resistance genes of antibiotic-resistant pathogens can improve current control strategies, which are based only on guidelines or ASPs. Such improved strategies can effectively intervene in prescribing on the basis of case-by-case scientific data.

Reducing antibiotic use in agriculture, especially in food animals, is also important. The problem of antibiotic resistance in human medicine cannot be solved unless the inflow of resistance genes into human microbiome, through food intake or contact with the environment, is restricted. To strengthen the immune system and promote the growth of food animals, various methods, including optimal usage of existing vaccines, improved hygiene, using health-improving enzymes, probiotics, prebiotics, and acids, and utilizing bacteriocins, antimicrobial peptides, and bacteriophages, as substitutes for antibiotics, should be given due consideration. Policies to change the use of antibiotics in agriculture can include ban or restriction on using medically important antibiotics, promising financial incentives for developing livestock-specific antibiotics, making drug licensing rules more stringent and imposing penalties on defaulters. Last, but the most important, strategy should be to invigorate the process of antibiotic development, so as to ensure uninterrupted availability of new weapons to combat antibiotic-resistant pathogens. Although the task of discovering novel targets of antibiotics is seemingly daunting, particularly with respect to Gram-negative bacteria, the quest must continue for innovative methods and policies.

**Figure 5:**
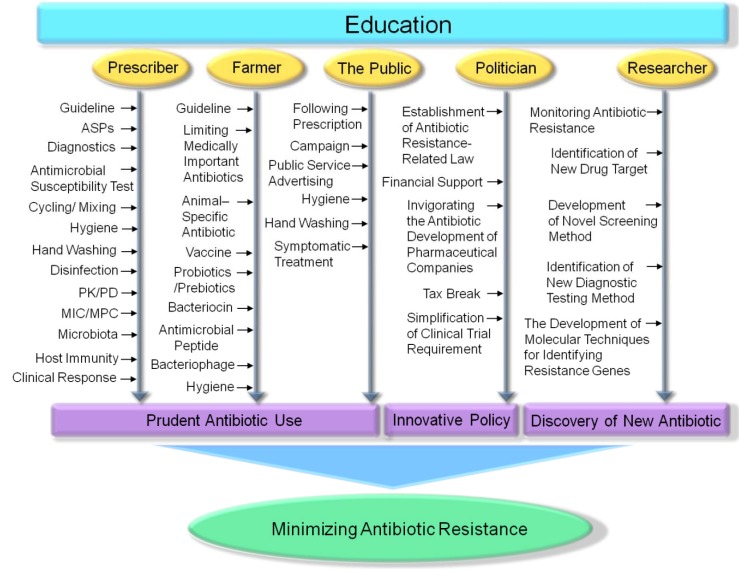
Strategies to minimize antibiotic resistance. Education is the very important strategy. The multidisciplinary core group, including physicians, pharmacists, microbiologists, epidemiologists and infectious disease specialists, can be the teachers educating various members of society. ASPs, PK/PD, and MIC/MPC mean antimicrobial stewardship programs, pharmacokinetic/pharmacodynamics properties of drug, and the minimum inhibitory concentration/the mutant prevention concentration, respectively.

In summary, concerted efforts of people involved in various fields, including prescribers, farmers, the public, politicians, and researchers, are needed to manage of antibiotic resistance (Figure 5). 

Above all things, continuous efforts to educate people about antibiotic resistance are the very important strategy. The multidisciplinary core group, including physicians, pharmacists, microbiologists, epidemiologists and infectious disease specialists, can educate various members of society. In hospitals, prescribers should use antibiotics, based on the recommendation of guidelines and ASPs, and through considering various data such as PK/PD and MIC/MPC of antibiotics, diagnostic testing results, AST results, clinical response, and effects on the microbiota. Thorough hospital disinfection and personal hygiene of healthcare workers, especially hand washing, are also important to prevent hospital-acquired infections. The guideline for the farmers should be quickly made. Farmers should not use medically important antibiotics such as carbapenems and vancomycin, and should consider the use of vaccines, bacteriocins, antimicrobial peptides, and bacteriophages as the alternatives of antibiotics. To strengthen the immune system in food animals, the utilization of enzymes, probiotics, prebiotics, and acids is a good choice. Improvement of hygiene on the farm is also meaningful. The public should prudently use antibiotics in accordance with prescriptions of physicians, and keep up personal hygiene such as handwashing and bathing. Public campaign and public service advertising are useful for clearing up the misunderstanding of the public about antibiotics. Politicians should establish antibiotic resistance-related laws and design innovative many policies to invigorate the development of novel antibiotics. Researchers should continue to monitor antibiotic resistance in hospitals, animals, and environments. Novel strategies for rational design and screening-based approaches are needed to discover new classes of antibiotics. The development of quick, effective molecular techniques for identifying resistance genes and the search of diagnostic biomarkers such as procalcitonin for using as a guide to cessation of antibiotics treatment are useful for reducing the use of antibiotics. Ultimately, if all members of society take on responsibility for maintaining the effectiveness of antibiotics and perform their role, minimization of antibiotic resistance can be successful.
